# SARS-CoV-2 Infections and COVID-19 Fatality: Estimation of Infection Fatality Ratio and Current Prevalence

**DOI:** 10.3390/ijerph17249290

**Published:** 2020-12-11

**Authors:** Marco Pota, Andrea Pota, Maria Luisa Sirico, Massimo Esposito

**Affiliations:** 1 Institute for High Performance Computing and Networking (ICAR)—National Research Council of Italy (CNR), 80100 Napoli, Italy; massimo.esposito@icar.cnr.it; 2 UOC Nefrologia e Dialisi Ospedale del Mare, 80147 Napoli, Italy; andrea.pota@gmail.com (A.P.); ml_sirico@yahoo.it (M.L.S.)

**Keywords:** COVID-19, SARS-CoV-2, case fatality rate, infection fatality ratio, prevalence

## Abstract

COVID-19 is one of the most important problems for public health, according to the number of deaths associated to this pathology reported so far. However, from the epidemiological point of view, the dimension of the problem is still unknown, since the number of actual cases of SARS-CoV-2 infected people is underestimated, due to limited testing. This paper aims at estimating the actual Infection Fatality Ratio (number of deaths with respect to the number of infected people) and the actual current prevalence (number of infected people with respect to the entire population), both in a specific population and all over the world. With this aim, this paper proposes a method to estimate Infection Fatality Ratio of a still ongoing infection, based on a daily estimation, and on the relationship between this estimation and the number of tests performed per death. The method has been applied using data about COVID-19 from Italy. Results show a fatality ratio of about 0.9%, which is lower than previous findings. The number of actual infected people in Italy is also estimated, and results show that (i) infection started at the end of January 2020; (ii) a maximum number of about 100,000 new cases in one day was reached at the beginning of March 2020; (iii) the estimated cumulative number of infections at the beginning of October 2020 is about 4.2 million cases in Italy (more than 120 million worldwide, if a generalization is conjectured as reasonable). Therefore, the prevalence at the beginning of October 2020 is estimated at about 6.9% in Italy (1.6% worldwide, if a generalization is conjectured).

## 1. Introduction

SARS-CoV-2 virus is one of the most infective viruses having emerged in the 21st century. Indeed, starting from China, as of end of October 2020, it has infected more than 40 million people around the world [1], acquiring the characteristics of a pandemic. The authors of an early study [2] suggested that SARS-CoV-2, although less fatal if compared to other coronaviruses (SARS-CoV and MERS-CoV), is dangerous for its far larger case count. In fact, its clinical expression, i.e., coronavirus disease (COVID-19), is one of the most important and actual problems for public health, according to the number of deaths associated to this pathology reported so far. As of end of October 2020, more than 1 million deaths emerge from data released online by World Health Organization (WHO) for public information [1], and this number is still increasing.

The worst aspect regarding this pandemic emergency consists in the lack of knowledge about this recently appeared problem. In fact, from the clinical point of view, treatments of serious cases have been developed very fast (e.g., [3,4,5,6,7,8,9,10,11,12,13,14,15,16]), and nowadays, they are more or less well established, but still a high number of deaths occurs, and a vaccine is still not available, since its implementation, if possible, still needs time to be completed. On the other hand, from the epidemiological point of view, the dimension of the problem is also still unknown, since the number of actual cases of infected people is underestimated, due to limited testing [17]; this hinders the accurate calculation of prevalence (number of infected people with respect to the entire population), Case Fatality Ratio (CFR, number of deaths due to COVID-19 with respect to the number of COVID-19 patients) and Infection Fatality Ratio (IFR, number of deaths due to COVID-19 with respect to the number of SARS-CoV-2 infected people).

This study takes into account the epidemiological side of the problem, the knowledge of which is needful to choose the most appropriate strategies to face the emergency. In particular, this paper aims at clarifying the following questions:

(i.) Which is the actual IFR, estimated as the number of deaths due to COVID-19 with respect to the number of SARS-CoV-2 infected people? IFR is studied instead of CFR, because most of the available data report all confirmed SARS-CoV-2 infected people as positive cases, without distinguishing between asymptomatic cases and COVID-19 patients, including subclinical and more serious cases.
(ii.) Which is the actual prevalence hereto, estimated as the number of (currently) infected people with respect to the entire population, both in a specific population and all over the world?

In order to answer these questions, a few numbers would be needed for a given population, i.e., the number of deaths, the number of infected people, and the size of the entire population. Given these numbers, IFR can be calculated and hypothesized as constant, while the prevalence is changing every day during the current period of ongoing infection. Among these numbers, the population size can be considered as known, while the others need more investigation.

The actual number of deaths due to COVID-19 is different from the number of confirmed cases, due to some sources of errors. First, the number of confirmed cases is overestimated, since it comprises people died after performing a laboratory test, but also people labelled as “probable”, without a test confirming their positivity. On the other hand, the number is underestimated, since some positive cases could have died without being hospitalized and labelled as confirmed COVID-19 cases. For example, in New York City, until 1 May 2020, 18,282 deaths were reported, of which 13,156 confirmed positive cases, and other 5126 probable cases [18]. On the other hand, a study [19] calculated, at the same date, an excess of 24,172 deaths with respect to the seasonal expected baseline.

The actual number of infected people is much more uncertain, and it is certainly underestimated by the number of confirmed cases [17], because there is a large number of asymptomatic cases, or people with symptoms mild enough to not require testing [3], and because the testing has not been applied on the entire population. For example, a study performing an antibody test on 15,000 healthy people [20] showed that in New York City, on 1 May 2020, 19.9% of the population had COVID-19 antibodies, which, multiplied by the entire city population (8,398,748 [21]), gives an estimation of 1,671,351 recovered cases, about ten times the number of confirmed cases at the same date (166,883 [22]).

Therefore, IFR, if calculated as the fraction of confirmed deaths with respect to confirmed infected people, is wrong for two main reasons. First, during a period when the infection is still ongoing, the calculation using current total numbers is misleading, due to the unknown outcome of people not yet resolved. Moreover, due to strong underestimation of positive cases, IFR is overestimated.

Some studies reported estimations of COVID-19 mortality obtained by dividing confirmed deaths by confirmed infected people. An early study reported 6 deaths over a limited cohort of 41 confirmed positive cases (CFR equal to 14.6%), and an overall IFR of about 3% in a wider sample in Wuhan [23]. However, authors stated, “both of these estimates should be treated with great caution because not all patients have concluded their illness (i.e., recovered or died) and the true number of infections and full disease spectrum are unknown”. Moreover, WHO reported, on 29 January 2020, “2% of confirmed cases are reported to have died” [24]. Later, on 28 February 2020, the WHO–China Joint Mission reported [25] that 2114 people died over 55,924 studied confirmed cases, which corresponds to an IFR of 3.8%. However, “The Joint Mission acknowledges the known challenges and biases of reporting crude CFR early in an epidemic”. The same report underlines dependence on risk factors and location. Finally, on 3 March 2020, WHO Director General stated, “Globally, about 3.4% of reported COVID-19 cases have died.” [26]. Moreover, the work that is the most recent to the best of our knowledge, reported a CFR of 7.05%, calculated by confirmed cases updated at 30 April 2020 [27]; this is actually an IFR estimate, since employed data include in confirmed cases all SARS-CoV-2 infected people, both COVID-19 patients or asymptomatic.

Other studies adjusted the calculation by only considering resolved cases, and calculating CFR as the number of deaths divided by the sum of deaths and recovered people, as suggested in [28]. For example, in [29], an IFR of 1.4% is found based on New York City adjusted data mentioned above, while values of 4% worldwide and 3.5% outside mainland China are found based on confirmed cases only [30]. However, this implies assumptions about the estimation of infected people.

An alternative method to calculate IFR, during an ongoing epidemic, is to consider daily cases, as suggested in [28]. In particular, in [31], statistical methods are employed to derive a time-delayed adjusted IFR estimate lower than 0.5%. However, this method (i) implies to obtain variable results every day, (ii) needs the knowledge of the time period between infection and resolution, and (iii) also implies assumptions about the estimation of infected people.

In this work, to answer the mentioned questions, available data regarding the situation in Italy have been employed. In particular, the actual number of deaths is considered here to be equal to daily reports released by the Civil Protection Department of the Presidency of the Council of Ministers of Italy. However, no assumption about the number of infected people is made. Instead, a novel method is developed to directly estimate IFR, and consequently estimate infected people and prevalence, by taking into account the relationship between the daily estimation of IFR and the number of tests performed per death, as detailed in the next section.

The possibility of generalizing outcomes based on an Italian data set to different situations is conditioned by the satisfaction of the following few hypotheses. Some of them may be reasonable in general, while others depend on the analysed situation. Firstly, a previous retrospective cohort study [32] allowed authors to conclude that black race was not associated with higher in-hospital mortality than white race. This finding suggests as feasible the hypothesis that in general the prognosis does not depend on the race of the patient, which is equivalent to hypothesize that the death rate does not directly depend on the prevalent race of the considered population. On the other hand, the disparities among population groups, regarding the limited accessibility to public healthcare system for ethnic minorities [33], could be more or less exacerbated with respect to Italy, depending on the socio-economic and cultural situation. Secondly, the hypothesis is needed that risk factors (e.g., those found in [3,4,32,34,35,36,37,38,39,40], and reviewed in [41]) are distributed in the considered population similarly to Italy. This is reasonable as long as situations are analysed where alimentary and lifestyle habits are comparable to Italy. Moreover, the generalization could be considered valid only for those situations where patients are treated similarly to Italy, thanks to similar clinical guidelines, and availability of drugs and room for hospitalization. Finally, further hypotheses are needed, like similarity with Italy in terms of climatic conditions, pollution, and maybe others based on still unknown influencing factors.

The rest of the paper is structured as follows: Section 2 describes the dataset and the methods used to estimate IFR and prevalence, while Section 3 presents and discusses results, and Section 4 remarks conclusions.

## 2. Materials and Methods 

### 2.1. Data

The data set used here regards the COVID-19 situation in Italy, and is released on GitHub [42] by the Civil Protection Department of the Presidency of the Council of Ministers of Italy. Data start from the recognized beginning of the infections, i.e., 24 February 2020, and are updated on a daily basis. Both the data about the entire nation and each region and province are included. However, for the aims of this work, only data about the whole Italy are considered. 

Numerous features are reported. They are the following: date, nation, hospitalized with symptoms, hospitalized with intensive care, total hospitalized, home isolation, total positives, variation of total positives, new positives, cumulative recovered, cumulative deaths, new cases found by suspected diagnosis, new cases found by screening, cumulative cases, cumulative tests, subjects tested, and notes. In this work, the following are used: date, total positives (SARS-CoV-2 currently positives), new positives (new SARS-CoV-2 positives at date), cumulative recovered (total SARS-CoV-2 former positives currently negative), cumulative deaths (total deaths due to COVID-19) and cumulative cases (total number of confirmed SARS-CoV-2 positive cases). Moreover, the further features new deaths (new deaths due to COVID-19 at date) and new tests (new tests performed at date) are simply calculated by daily increments of cumulative deaths and cumulative tests, respectively. In Table 1, a sample of data used here is reported, in correspondence of some notable dates (the first two days reported, the day with highest number of deaths, the last day of the lockdown, and the most recent date available).

### 2.2. Existing Methods for Estimating IFR 

As mentioned in the Introduction, different methods exist to estimate CFR, and similarly IFR.

The first method simply divides the number of deaths due to COVID-19 by the total number of confirmed SARS-CoV-2 positive cases [28]:

(1) $$IFR_{1}=\frac{cumulative\;deaths}{cumulative\;cases}.$$

However, this method gives a wrong estimation, because it could not be applied while the infection is still ongoing [28], and because actual cumulative cases are surely underestimated by confirmed cumulative cases.

A second method allows mitigating the first problem, by only taking into account resolved cases (comprising deaths due to COVID-19 and recovered cases). It compares the number of deaths with the number of resolved cases [28]:

(2) $$IFR_{2}=\frac{cumulative\;deaths}{\left(cumulative\;deaths+cumulative\;recovered\right)}.$$

However, also in this case, the estimation of actual cumulative recovered is not trivial.

### 2.3. Proposed Approach for Estimating IFR and Prevalence

The proposed approach consists in a two-step procedure. In the first step, IFR is estimated on a daily basis. In the second step, the functional relationship is assessed between daily estimates and the ratio of daily tests performed with respect to the number of new cases, and the limit of IFR is estimated as the ratio approaches infinity.

The initial step starts from a third method to calculate IFR, suggested in [28]. It calculates IFR_1_ on a daily basis: similarly to IFR_1_, where cumulative deaths at the end of the infection are a fraction of cumulative cases, during the infection, the new deaths each day should be the same fraction of the new positives of some days before:

(3) $$IFR_{3}=\frac{new\;deaths\left(i+T\right)}{new\;positives\left(i\right)},$$

where *i* is the variable date and *T* is the medium number of days from infection to fatal resolution.

With this method, a definition of *T* is needed. Luckily, two studies on the development of COVID-19 have shown that, for non-survivors patients, death occurs on average during the 19th day after the first symptoms and beginning of inspections [43], while for survivors, the average time from onset of symptoms to negative testing is 19 days [43,44]. Moreover, another study estimated the median period from virus exposure to first symptoms onset in between 5 and 6 days [45]. Therefore, here, the following *T*, obtained by summing up the two periods between exposure and death, is chosen:

(4) T=24.

Actually, the time period between infection and eventual death is variable; however, here, this time delay is fixed at its median value, according to literature findings. Results will be shown both according to this fixed value of *T*, and as it varies between 1 and 40 days.

On the other hand, this method also needs to estimate actual positive cases. However, while other methods need to estimate cumulative cases, this one needs new positive cases.

As the second step of the approach, here is proposed to employ the obvious observation that the estimate of positive cases is better the more tests are performed. However, actual cases are not estimated at first based on tests, since they are intrinsically variable; moreover, the number of tests performed could be biased itself by the trend of infections.

Instead, IFR is directly estimated. In fact, IFR can be hypothesized as constant, during a period of constant strategies of treatment for patients; therefore, its variations during time should be mainly due to underestimation of positive cases. In particular, IFR is estimated according to (3):

(5) $$IFR=\underset{\frac{new\;tests\left(i\right)}{new\;deaths\left(i+T\right)}\to +\infty }{\lim}\frac{new\;deaths\left(i+T\right)}{new\;positives\left(i\right)}.$$

The measure that should tend to infinity is chosen for these reasons:

(i.) obviously, the estimate of new positives on day *i* is better the more tests are performed on day *i*;
(ii.) the quantity of tests should not be evaluated as an absolute value, but with respect to the number of actual positive cases;
(iii.) the number of actual positive cases on day *i* is unknown, but according to Equation (3) it is proportional to new deaths on day *i* + *T*.

The limit can be evaluated on the basis of the available data. The IFR values approaching the asymptote can be chosen as those corresponding to the highest tests/deaths ratio. In particular, the mean of the last three values is considered here, to average errors due to stochastic deviations.

Once IFR is estimated, actual positive cases can be estimated inversely:

(6) $$actual\;new\;positives\left(i\right)=\frac{new\;deaths\left(i+T\right)}{IFR},$$

(7) $$actual\;cumulative\;cases=\frac{cumulative\;deaths}{IFR},$$

Consequently, the prevalence can also be calculated:

(8) $$Prevalence=\frac{actual\;cumulative\;cases}{Population},$$

Of course, cumulative positive cases and prevalence are still variable during ongoing infection; therefore, their values have to be associated to the corresponding date when they are calculated.

## 3. Results and Discussion

In this section, data described in Section 2.1 are used. Results presented regards firstly classic methods reported in Section 2.2, and finally the proposed approach detailed in Section 2.3.

**Figure 1:**
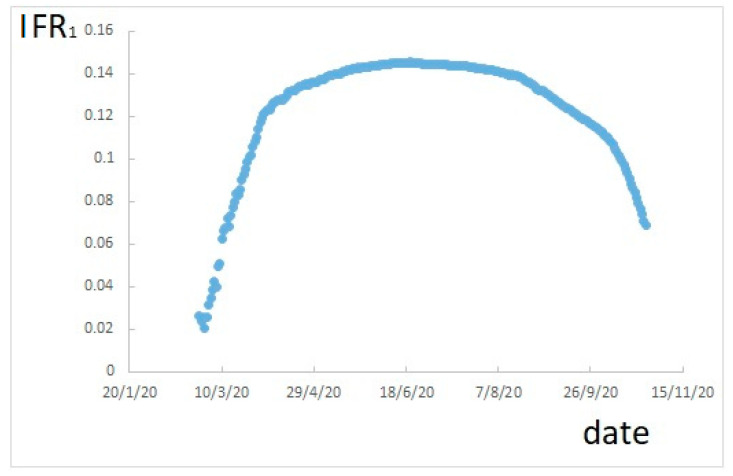
Infection Fatality Rate (IFR) calculated as cumulative deaths divided by cumulative confirmed positive cases.

According to Equation (1), IFR_1_ varies during the ongoing infection. For the Italian case, it would result as shown in Figure 1.

Figure 1 shows that, during the infection, the rate of fatalities changes. Moreover, a maximum can be observed in correspondence of a period with a relatively low number of new cases, which should be similar to the situation at the end of the infection. However, the maximum value of 14.5% is certainly overestimated, due to the underestimation of actual positive cases.

**Figure 2:**
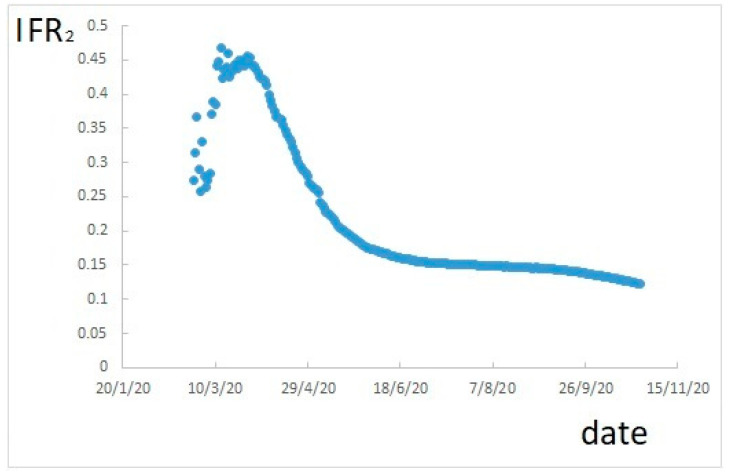
IFR calculated as cumulative deaths divided by cumulative resolved cases.

If IFR_2_ is calculated, according to Equation (2), it results as shown in Figure 2.

Figure 2 also shows a variability. In this case, a maximum is reached in a very early period, probably due to the fact that, at the initial stage, only patients with severe symptoms were detected as positive. Therefore, the value of the maximum is hardly interesting. A plateau is reached around 15%, but it is still decreasing. However, this estimation also is overestimated due to the underestimation of positive cases.

**Figure 3:**
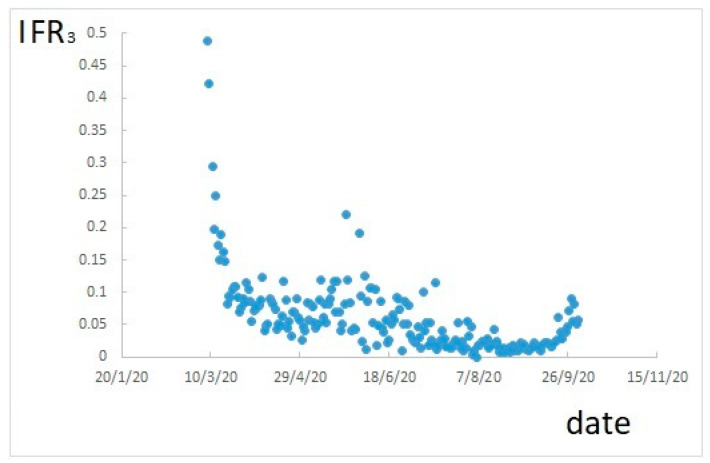
IFR calculated as daily deaths divided by positive cases confirmed 24 days before.

If IFR_3_ is calculated, according to Equation (3), it results as shown in Figure 3.

**Figure 4:**
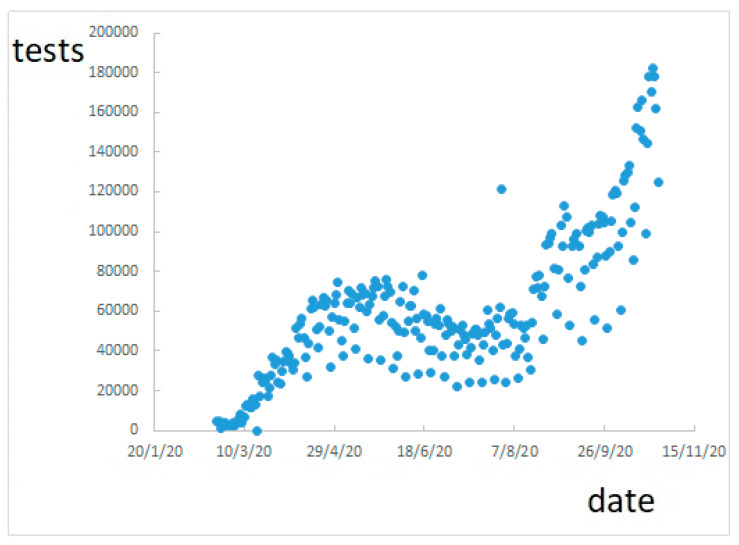
Number of test performed.

Figure 3 shows still a variable trend, and underestimates positive cases. However, this trend is much correlated to that of the number of tests performed, shown in Figure 4 (compare Figure 3 and Figure 4).

**Figure 5:**
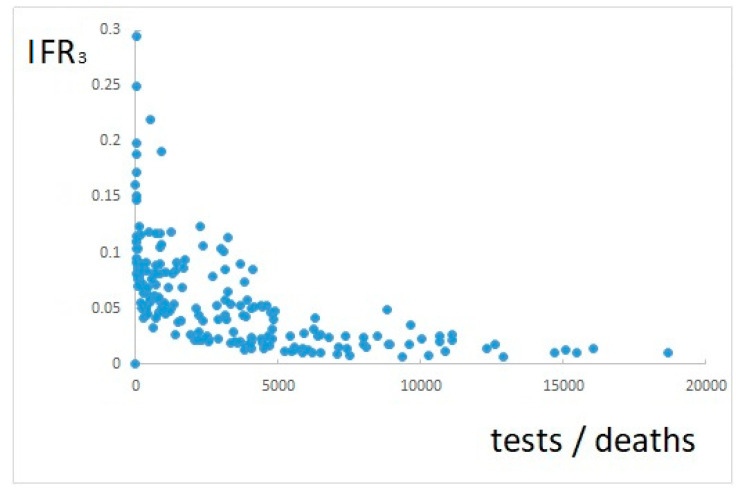
IFR with respect to the scaled number of tests performed.

The approach proposed here consists in evaluating IFR, calculated according to Equation (3), with respect to the number of tests performed divided by the number of deaths, as detailed in Section 2.3. This evaluation is based on data plotted in Figure 5.

Figure 5 shows a clear asymptotical decreasing trend, which confirms the suitability of the proposed approach. IFR can be estimated by Equation (5), i.e., by the values of IFR_3_ at the right end of the figure, approaching the asymptote, as explained in Section 2.3. This ultimately corresponds to an IFR estimate of about 0.9%.

**Figure 6:**
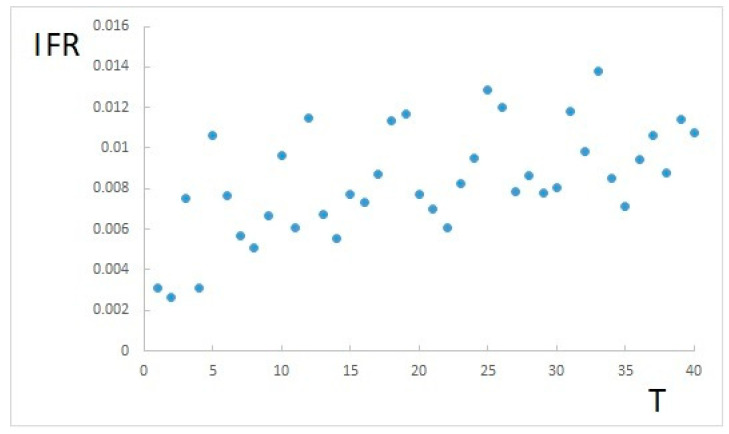
IFR with respect to the average time between infection and death.

Since the present study allows just estimating IFR, which results in a value of about 0.9% for *T* = 24, its range of possible actual values is also reported. In order to evaluate how the estimated value depends on the time between infection and death, *T*, the same method applied for *T* = 24 (obtained by literature findings) is also executed for different values of *T*. Figure 6 shows how IFR varies accordingly.

From Figure 6, a certain range of variation of IFR can be inferred, depending on the chosen *T*. These variations appear to be mostly random, due to data distribution, and slightly dependent on *T*. However, most of the values between *T* = 5 and *T* = 40 are within the same range. Therefore, it can be concluded that, even if *T* could be actually different from literature findings (*T* = 24), IFR is nonetheless in the range of 0.6% to 1.3%.

**Figure 7:**
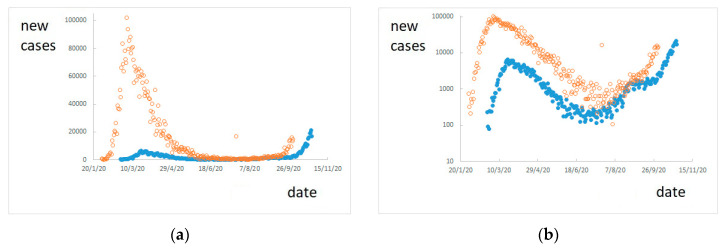
Confirmed new cases (blue full dots) and estimated actual new cases (orange empty dots): (**a**) in linear scale and (**b**) in logarithmic scale.

Using the estimated value of IFR, the number of daily actual new positive cases can be estimated by Equation (6). In particular, using the IFR value of 0.9%, the trend of actual new cases is reported in Figure 7, together with the trend of confirmed new cases. 

Figure 7 shows estimated new cases translated in time with respect to known confirmed cases, because the estimation regards new cases occurred *T* = 24 days before known deaths, i.e., when presumably they have been infected, while confirmed cases have been detected some days after their infection. In particular, according to the hypotheses made here and to the available data, the spread of the infection in Italy seems to have started at the end of January 2020 (about 24 days before the first reported death on 21 February).

Another interesting piece of information that can be inferred from Figure 7 is that the actual number of new cases is at least ten times the confirmed new cases, during the first period of infection, i.e., until August 2020. Later, the high number of tests performed has allowed detecting a number of new cases more similar to the actual number.

The estimated number of new cases shown in Figure 7 presents a maximum of about 100,000 new cases in one day, on 3 March 2020, corresponding to the maximum of 969 deaths in one day on 27 March.

Moreover, using the estimated value of IFR, the actual cumulative number of cases (died, recovered, or still positive) can be calculated by Equation (7). It refers to the cumulative number of deaths reported on 26 October 2020 (37,479); therefore, it is valid as an estimate regarding the date 2 October 2020. The actual cumulative number of cases on that date is about 4.2 million cases, more than ten times the confirmed cases (319,908).

If results found here are generalized, by speculating that the conditions of similarity between Italy and the rest of the world may be reasonably valid, a number of more than 120 million cases of SARS-CoV-2 infections worldwide is found, according to 1,114,749 deaths reported on 18 October 2020 [46].

Finally, using the estimated number of cases, the prevalence of SARS-CoV-2 infections can be calculated by Equation (8). On 2 October 2020, the prevalence in Italy results about 6.9% (1.6% worldwide if the generalization is conjectured as reasonable). These values are much higher than those expected from confirmed cases. However, this prevalence is still much too low to reach the target of around 67% needed for herd immunity.

## 4. Conclusions

This paper proposes a method to estimate Infection Fatality Ratio during a still ongoing epidemic, based on daily estimates, and on their relationship with the number of tests performed per death. Consequently, the method has been applied on data regarding SARS-CoV-2 situation in Italy. Results show a fatality rate of about 0.9%, which is lower than previous findings. The number of actual positive cases in Italy is also estimated, and results show that (i) infection started at the end of January 2020; (ii) a maximum number of about 100,000 new infections in one day was reached at the beginning of March 2020; (iii) the estimated cumulative number of infections at the beginning of October 2020 is about 4.2 million cases in Italy (more than 120 million worldwide, if a generalization is conjectured as reasonable). Therefore, the prevalence at the beginning of October 2020 is estimated at about 6.9% in Italy (1.6% worldwide if a generalization is conjectured). We are still very far from herd immunity.

## Figures and Tables

**Table 1:** Examples of daily data used.

Date	Total Positives	New Positives	Cumulative Recovered	Cumulative Deaths	New Deaths	Cumulative Cases	New Tests
24 February 2020	221	221	1	7	7	229	4324
25 February 2020	311	93	1	10	3	322	4299
27 March 2020	66,414	5959	10,950	9134	969	86,498	33,019
02 June 2020	39,893	318	160,092	33,530	55	233,515	52,159
22 October 2020	169,302	16,079	259,456	36,968	136	465,726	170,392
